# Situated Student Sense of Belonging in Higher Education: The Importance of Context and Engagement

**DOI:** 10.3390/bs16101809

**Published:** 2026-10-02

**Authors:** Simon Cassidy, Clare Edge

**Affiliations:** Faculty of Health, Community & Life Sciences, University of Salford, Frederick Road, Salford M6 6PU, UK; c.e.edge1@salford.ac.uk

**Keywords:** student sense of belonging, departmental context, higher education, minoritized ethnic, student engagement, student involvement

## Abstract

While the benefits of student sense of belonging in higher education are well established, lower levels of belonging among minoritized ethnic students and fluctuations over time are frequently reported. However, most research focuses on the wider institution, overlooking the department as the primary setting for meaningful interpersonal interactions that foster belonging. Using students’ ‘home’ department as the principal context for belonging, this longitudinal study employed psychometric measures across the first year of study to examine situated sense of belonging, exploring differences by ethnicity and changes in belonging over time. Relationships between belonging, involvement, and engagement were also explored. Contrary to previous findings, ethnicity had no effect on belonging. However, both belonging and involvement declined over time, whereas engagement remained stable. Engagement, but not involvement, emerged as a significant predictor of belonging. The absence of previously reported differences according to ethnicity highlights the importance of context, supporting the situated nature of student belonging and suggesting that departments may have greater potential than institutions to foster belonging, mitigate inequality, and support student wellbeing and success. The reported decline in students’ belonging signals the need for sustained interventions that promote engagement and belonging beyond the initial stages of the students’ entry into higher education.

## 1. Introduction

Early work exploring student dropout in higher education recognized the importance of institutional commitment (i.e., students’ predisposition, loyalty and commitment to a [their] particular university) and the role of friendship support, social integration, and sense of belonging (Nora & Cabrera, 1993; Spady, 1971; Tinto, 1975). As universities continue to focus on improved student wellbeing, inclusivity, attainment, and retention (Hillman, 2024), student sense of belonging has emerged as a ‘hot topic’, considered critical for a positive student experience in higher education and a negative predictor of students’ intention to leave (Allen et al., 2024; Crawford et al., 2024; Gilani & Thomas, 2025). Described as ‘‘the subjective feeling of deep connection with social groups, physical places, and individual and collective experiences’’, there is general acceptance that a sense of belonging is a fundamental human need affecting motivation and predictive of outcomes (Allen et al., 2021, p. 1). As a “vital” mental health concept (Hagerty et al., 1992) and a need rather than a desire (Baumeister & Leary, 1995; Maslow, 1954), failure to satisfy a sense of belonging leads to negative outcomes and long-term negative consequences (Over, 2016), with the association between sense of belonging and student wellbeing and success considered robust (Gilani & Thomas, 2025).

Findings from studies conducted in higher education further support the benefits of student sense of belonging, including greater motivation, academic self-confidence, enjoyment, academic engagement, persistence, achievement, and wellbeing (Allen et al., 2021). Similarly, evidence garnered from research conducted in schools supports a positive association between student sense of belonging and student enjoyment, effort, persistence, self-efficacy, achievement motivation, social acceptance, attendance, attainment, and wellbeing, along with a reduced risk of dropping out (Slaten et al., 2018). Summarizing the evidence relating to sense of belonging and student retention and success, Crawford et al. (2024, p. 396) note that “when people belong, they stay”. This highlights a clear imperative for higher education institutions to commit to fostering a strong sense of belonging in their students and the need for continued research exploring conceptualization, measurement, and critical factors associated with sense of belonging. Central to this is gaining insight into how belonging changes in students over time (i.e., its temporal trajectory), the effect of student ethnicity, and the role of student involvement and engagement.

## 2. Literature Review

### 2.1. Conceptual Issues

Described by Allen et al. (2021) as the most common definition, Goodenow and Grady (1993, pp. 60–61) define sense of belonging in schools as “the extent to which students feel personally accepted, respected, included, and supported by others—especially by teachers and other adults in the school social environment”. Definitions of student sense of belonging in higher education typically include the concepts of acceptance, connectedness, support, valuation, respect, inclusion, importance, mattering, feeling or being part of, and fitting in, while student definitions add feeling capable, recognized, acknowledged, safe, similar, and a need for self-investment and wanting to contribute. Focusing specifically on higher education, Strayhorn (2019, p. 4) describes sense of belonging as referring to “students’ perceived social support on campus, a feeling or sensation of connectedness, and the experience of mattering or feeling cared about, accepted, respected, valued by, and important to the campus community or others on campus such as faculty, staff, and peers”.

Despite common characteristics and broad agreement, there remains a lack of consensus regarding the conceptual definition and measurement of student sense of belonging. This is highlighted as a particular issue for studies in higher education, where research is described as “sporadic and inconsistent” (Slaten et al., 2018, p. 634). A recent review of 150 studies of sense of belonging in higher education identified over fifty different sense of belonging measures, found evidence that these studies were using a range of different concepts to define sense of belonging, and reported misalignment between definitions and measures of sense of belonging adopted in studies as common (Dias-Broens et al., 2024). Helpful in this regard, Dias-Broens et al. (2024) propose a typology of the higher education literature, locating the facets of student sense of belonging according to three separate underlying aspects: concepts, social entities, and context. Similar to Allen et al.’s (2021) account of belonging defined in terms of connection with *people*, *places*, and *experiences*, ‘social entities’ include groups, peers, teaching and non-teaching staff, and campus community, while ‘contexts’ include institution, campus, classroom, and academic course or program. Importantly, social entities and contexts help define groups, places, and contexts where students can attach sense of belonging, acknowledging the *situational* nature of sense of belonging whereby perceived connectedness or mattering can vary according to different social entities and contexts (Dias-Broens et al., 2024). For instance, peers versus staff, department versus institution. As Allen et al. (2021, p. 3) describe it, “belonging is facilitated and hindered by people, things, and experiences of the social milieu, which dynamically interact with the individual’s character, experiences, culture, identity, and perceptions.”

### 2.2. Minoritized Students

Like other psychological concepts, self-efficacy, for instance, the significance of sense of belonging becomes more evident under certain conditions. Strayhorn (2019, p. 5) argues that sense of belonging becomes increasingly important “in *certain* social contexts where *some* individuals are prone to feeling unsupported, unwelcomed, or lonely, or in *some* social contexts where *certain* individuals are more likely to feel that way”. Using first-year students in a new university as an example, Strayhorn (2019, p. xiv) suggests that the need for a sense of belonging becomes “heightened” in unfamiliar “contexts and settings where individuals are prone to feel alienated, invisible, (pre)judged, stereotyped, or lonely”—or marginalized in some way. Allen et al. (2021, p. 3) offer support for Strayhorn, pointing out that “struggles to belong are particularly evident in minorities and other groups that have been historically excluded from mainstream culture”.

Minoritized ethnic students are described as those students who attend an academic institution in a country where the majority of people have a different ethnicity to that of the student (Dias-Broens et al., 2024). Yorke (2016) compared sense of belonging between White British and minoritized ethnic students in thirteen separate higher education institutions, reporting higher sense of belonging in White British students in each of the included institutions. Such evidence that minoritized ethnic students report lower sense of belonging than non-minoritized ethic students is concerning (Dias-Broens et al., 2024), particularly given that pupils from ethnic minorities are more likely than their White British counterparts to progress to university (Martin, 2025). These adverse differences in sense of belonging in university students according to race and ethnicity highlight the importance of identifying and understanding factors with the potential to mitigate differential degree outcomes and contribute to reducing ‘the degree award gap’ and poor retention rates (Arday et al., 2021; Universities UK, 2022). In a study involving non-continuing ethnic minoritized university students, Kauser et al. (2021) reported social integration and peer support as salient factors in reduced sense of belonging and confidence in students to attend lectures and seek support. Similarly, in their review of belonging in marginalized students in higher education, Taff and Clifton (2022) identify social exclusion as the most common barrier to sense of belonging. Less academic staff contact and support and increased staff stereotyping have also been identified as factors explaining lower sense of belonging in Students of Color compared with White students (Kim & Lundberg, 2026).

Not all evidence is consistent with differences in sense of belonging between minoritized ethnic and non-minoritized students. In one study where ethnicity did not influence students’ sense of belonging, the study’s authors highlight that the study was conducted during the COVID-19 pandemic, which they assert may explain the contradictory findings (Spaducci et al., 2024). Other studies report more mixed results, where the pattern of the association between ethnicity and sense of belonging is more complex and related to aspects of intersectionality (Ovink et al., 2024).

### 2.3. Temporal Trajectory

The limited scope of research exploring changes over time (i.e., temporal trajectory) in student sense of belonging, particularly in higher education, is widely acknowledged (e.g., Ruedas-Gracia et al., 2023). However, based on (i) Allen et al.’s (2024) argument that students’ sense of belonging may evolve overtime as a result of changing environments and personal development experienced during their academic journey, (ii) evidence of fluctuations in student sense of belonging overtime (Gillen-O’Neel, 2021; Park et al., 2012), and (iii) longitudinal studies reporting declining student sense of belonging during the first year in higher education (Gilani et al., 2026; Hausmann et al., 2007) and steady decline from first to final year, with a steeper drop observed for minoritized ethnic students compared to White students (Brown & Papp, 2025), temporal variation in students’ sense of belonging might be expected. However, not all studies report significant changes over time. Two recent studies, Barringer et al. (2023) and Ruedas-Gracia et al. (2023), report stable levels of sense of belonging over time in university students demonstrated across two and three time points, respectively. However, Barringer et al. (2023) do report a moderating effect of ethnicity, with a significant decline in sense of belonging in minoritized ethnic students but not White students, while Ruedas-Gracia et al. (2023) note significant nuanced differences in temporal trajectories according to student ethnicity, sex, and income. Both studies were, however, conducted, in part, during the COVID-19 pandemic and included at least some data collection points pre-, during, or post-lockdown and associated remote learning conditions. This may have acted as a confounding factor, making it difficult to draw conclusions regarding the typical temporal trajectory of student sense of belonging in higher education based on these studies.

Having measured belonging specifically in the context of the wider university campus community, Brown and Papp (2025) explain the decline in student sense of belonging in terms of ‘de-identification’, whereby students become increasingly more inclined to identify with their departmental communities as they progress in their academic journey. A lack of ‘identity salience’ explains the decline in sense of belonging through a process of de-identification with the wider university campus community. This highlights the importance of accounting for context in student sense of belonging studies, raising the possibility that studies focusing on department sense of belonging would reveal a different temporal trajectory to those focusing on the wider university context. Accordingly, Brown and Papp (2025) call for more studies focused on student sense of belonging at the department level, exploring how to foster and maintain sense of belonging over time.

### 2.4. Situated Student Sense of Belonging

Critical to the development of sense of belonging are interpersonal interactions (i.e., ‘connections’) (Baumeister & Leary, 1995; Strayhorn, 2019). These connections are concrete acts “situated in time and space” (Ajjawi et al., 2026, p. 199). Key then is the argument that the greatest opportunities for meaningful student-student/student-staff interactions, as well as gaining knowledge, confidence and identity related to students’ interests and goals at university, exist in students’ ‘home’ department where the students’ academic program is situated (Knekta et al., 2020). Their department is where students are expected to dedicate a significant proportion of their time, energy, and attention while both on campus and in virtual learning environments. Yet there is limited available evidence from situated student sense of belonging research conducted in the context of students’ home department. Instead, studies frequently emphasize the wider academic institution as the primary context in which to measure students’ perceptions of belonging (Knekta et al., 2020). Dost and Smith (2023, p. 823) refer to students feeling that they are “part of the university”, while Hurtado et al. (2007, p. 856) focus on institutional-level community and refer explicitly to the institution in their definition of student sense of belonging: “the extent to which the student felt part of the campus community, saw him/herself as a member of the college, and had a strong sense of belonging at his or her respective institution”. Similarly, Tinto (1975, p. 90) describes his own student integration model as “institutionally oriented”. Tinto (1997, p. 613) did, however, later recognize how program-level learning leads to the development of ‘small supportive community of peers’, helping students engage with the ‘broader social communities [and] academic life of the institution’. Sense of belonging measures also favor the institution (i.e., school, college, university) as the context for measurement (e.g., E. Cohen & Viola, 2022; Goodenow, 1993; Yorke, 2016).

The limited attention given to students’ department as the primary context for studies is surprising given the dynamic situational nature of sense of belonging (Allen et al., 2021; Dias-Broens et al., 2024). It has, it is argued, resulted in a dearth of the most meaningful evidence, limiting understanding of student sense of belonging in higher education. This is further emphasized by Kern et al.’s (2020, p. 709) assertion that “humans interdependently co-exist with themselves, others, and the environment in which they exist [and] because of and in connection with the systems in which we reside”. Thus, the importance of the department, where students ‘reside’, should not be overlooked. Acknowledging that “sense of belonging in a certain context has the greatest influence on outcome in that particular context” and pointing out that departments are better positioned to respond and alter practice to more directly address issues around student sense of belonging based on relevant, department-level, insight, Knekta et al. (2020, p. 2) developed the Departmental Sense of Belonging and Involvement questionnaire (DeSBI) as a measure of *situated* student sense of belonging. Knekta et al. (2020) offer evidence supporting the reliability and validity of the DeSBI, identifying differences between student groups and recommending it for use in studies exploring fostering sense of belonging and identifying areas for improved practice in university departments.

### 2.5. Student Involvement and Engagement

As well as being important for student success in higher education—Bujold et al. (1997) refer to accounts of student involvement describing it as one of the “conditions of excellence for higher education [and] perhaps the most important for improving undergraduate education” (p. 26)—student involvement and student engagement have been closely associated with student sense of belonging (Osterman, 2000; Strayhorn, 2019; Thomas, 2012). Finn’s (1993) participation-identification model of student involvement and engagement proposes that, mediated by student success, participation in class and extracurricular activities helps students develop a sense of belonging. Astin (1999) proposes a student involvement theory where greater student involvement is associated with increased learning and development, such that “more is better” and defines student involvement as ‘the quantity and quality of the physical and psychological energy that students invest in the college experience’ (p. 528), with a focus on student behavior and motivation rather than the subject matter or techniques for learning. Astin (1999) further describes three types of involvement as *academic involvement*, defined as “the extent to which students work hard at their studies, the number of hours they spend studying, the degree of interest in their courses, good study habits” (p. 525), *involvement with peers*, and *involvement with faculty* (i.e., student–faculty interaction). These closely align with academic and interpersonal aspects of sense of belonging proposed by Nunn (2021) and other authors, while Bujold et al. (1997) add that affect, behavior, actions, and future-oriented intentions are all constituents of types of involvement.

Although the critical nature of student engagement and its association with student sense of belonging is widely reported, there is considerable variation in how engagement is defined and used in different contexts (Kahu & Nelson, 2018). The four perspectives or approaches to engagement have been identified as behavioral, psychological, sociocultural, and holistic (Kahu, 2013). The focus of these approaches widens gradually from student behaviors, broadening to include behavioral, cognitive, and affective aspects—the student’s “psychosocial state” (Kahu & Nelson, 2018, p. 59)—to acknowledging the wider socio-political context influencing aspects of engagement, to an integration of all the various engagement perspectives. A further challenge arises from the use of involvement and engagement interchangeably, even as synonyms, in the literature (e.g., Finn, 1993). This close and somewhat ambiguous relationship has been noted by Bujold et al. (1997), who suggest that the two concepts can be distinguished, in part, in terms of varying intensity, with engagement, preceded by involvement, representing greater intensity. Similarly, Bowden et al. (2021, p. 1211) note the emerging account of student engagement as a multidimensional meta-construct encompassing behavioral, affective, cognitive, and social engagement as distinct interrelated dimensions, with student involvement “motivating one’s attention and commitment” identified as an antecedent (“precondition”) for positive student engagement, “driving” behavioral engagement. According to Korhonen et al. (2024, p. 2), *behavioral* engagement relates to students’ “tangible participation”, cognitive engagement to ‘investment in learning’, and *emotional* engagement to “emotional responses in the context of learning and interaction with peers”. Behaviorally engaged students are described as exhibiting “proactive participatory behaviors through their involvement and participation in university life, and extracurricular citizenship activities” (p. 1212). Extending Kahu and Nelson’s (2018) argument regarding process and outcome facets of engagement, and Astin’s (1999, p. 519) emphasis on behaviors (i.e., what the student ‘does’) as the defining feature of involvement, involvement may be better viewed as representing the ‘process’ influencing engagement, with student engagement as the ‘outcome’. The connection between behavioral engagement and involvement has been suggested previously, where behavioral engagement is “seen as a direct indicator of involvement in learning” (Maroco et al., 2016; Otermans et al., 2025, p. 2). Behavioral engagement has also been referred to as “physical engagement”, defined as students’ effort (time and energy) studying and completing tasks (Sun et al., 2024, p. 175).

As definitions and measures of involvement and engagement dimensions vary widely, so do findings from studies conducted in higher education. Collecting data weekly during one term, Boulton et al. (2019) report a significant downward trend in involvement, with students’ participation in learning and extracurricular activities, including attending lectures, accessing the library, going to clubs and societies, and using sports facilities, all falling across the weeks. Boulton et al. (2019) suggest this could be due to students’ eagerness to ‘get ahead’ at the start of term giving way to assessment and associated demands faced by students later in the term. Exploring the effects of artificial intelligence (AI) tools on learning and measuring engagement over the course of a single semester, Bognar and Khine (2025) report a significant drop in student engagement at the end of the semester compared to the beginning, suggesting that initial levels of engagement are due to enthusiasm prompted by novelty, which then diminishes as students become more familiar with the AI tool approach. In contrast, Korhonen et al. (2024) and Saqr and Lopez-Pernas (2021) each report a stable temporal trajectory for student engagement. In a large-scale longitudinal follow-up study involving thirteen universities, Korhonen et al. (2024) reported that student engagement remained stable across the three years of the academic program, while Saqr and Lopez-Pernas (2021) reported between, but not within, student differences in engagement in a four-year study. That both of these studies measured engagement in the broader sense, capturing cognitive, affective, and social aspects as well as behavior, may explain why findings differed from studies such as Boulton et al. (2019), where the focus was on student involvement and was limited to measuring student behaviors. In fact, despite reporting an overall decline in engagement, Bognar and Khine (2025) do also report stable engagement (cognitive/affective/social) in those students who continued to interact frequently with AI learning tools, supporting a positive association between involvement/behavioral engagement (i.e., use of AI tools) and the broader aspects of engagement as suggested by Bowden et al. (2021).

Although equality, diversity, and inclusion remain a priority for higher education, few studies specifically investigate differences in involvement and engagement according to student ethnicity in higher education. One exception is Otermans et al. (2025), who compared measures of three engagement dimensions (behavioral, cognitive, and emotional) between White, Asian, and Black undergraduate first- and second-year students using the University Student Engagement Inventory (USEI) (Maroco et al., 2016). Despite reporting ethnicity as a significant factor in students’ preferences for types of assessment, no differences between ethnic groups were found in relation to any of the engagement dimensions.

### 2.6. The Present Study

Informed by complementary theoretical perspectives, models, and frameworks of student belonging, involvement, and engagement in higher education that emphasize social entities, context, integration, identification, commitment, and an evolving nature, the study conceptualizes sense of belonging as dynamic, context-specific, and dependent on students’ interactions with their immediate academic environment. This perspective suggests that students’ sense of belonging is situated, such that opportunities for involvement and engagement within students’ home department are particularly meaningful and, we argue, key contributors to its development. Although the importance of context and situation in shaping sense of belonging has been acknowledged, it has been somewhat neglected in higher education research, and there remains limited evidence from studies conducted (situated) in students’ home department. Focusing on students’ first year of university as a critical period for the development of sense of belonging and success (Ajjawi et al., 2026; Dias, 2022), the study explores differences in student sense of belonging, situated in the context of a student’s home department, according to ethnicity and time. The relationship between situated student sense of belonging and both student involvement and student engagement as contributory factors is also explored. Seeking to provide further evidence and insight regarding the proposed situated nature of sense of belonging in higher education, the study addressed three principal questions: whether previously reported differences in student sense of belonging between minoritized ethnic and non-minoritized ethnic students are also evident in the context of students’ home department; whether student involvement and student engagement are associated with situated student sense of belonging in this context; and whether situated student sense of belonging and its relationship with involvement and engagement alters during students’ first year at university. Based on existing evidence, if sense of belonging situated in students’ home department reflects sense of belonging in other contexts, such as to the wider university or institution, it is anticipated that non-minoritized students will report significantly greater sense of belonging than minoritized ethnic students, and that there will be a significant positive association between situated student sense of belonging and both student involvement and student engagement. Though limited, existing evidence suggests that differences in student involvement and student engagement according to student ethnicity are not expected, while a decline or fluctuation over time in student sense of belonging and student involvement, but not student engagement, is expected.

Using a psychometric questionnaire-based survey that includes the DeSBI (Knekta et al., 2020) and a separate student engagement scale developed by Yorke (2016) completed at three points during students’ first year, the study seeks to provide much-needed insight into the development and temporal trajectory of situated student sense of belonging, with a particular focus on differential effects according to ethnicity and time. Such insight will lead to an improved formulation of the sense of belonging concept, informing the development of more precisely targeted interventions aimed at developing and maintaining student sense of belonging in higher education. Two additional student sense of belonging measures are included to further establish the validity of the DeSBI and to ensure that the study captures aspects of sense of belonging that may resonate particularly with marginalized students, and that may not be fully addressed by other measures. These are the Sense of Belonging Scale (E. Cohen & Viola, 2022), an existing measure developed at Imperial College London, and the Brief Departmental Sense of Belonging Scale (Brief DSSoB) developed for the purpose of the present study.

## 3. Method

### 3.1. Design

The study adopted a repeated-measures, single-group dynamic (open) cohort longitudinal design using an anonymous online psychometric survey. The design allows participants to join the study at any of the data collection points, helping mitigate high participant attrition rates commonly reported in longitudinal studies (Bamer et al., 2020). The survey included three separate self-report student sense of belonging measures incorporating a student involvement subscale and a separate self-report student engagement measure. Demographic questions (age, gender, ethnicity, and academic program) were presented at the end of the survey. Partial counterbalancing, where the order of measures presented in the survey was rotated, was used in trimester three to help minimize order effects. The survey was administered at three data points across students’ first year of study, once per trimester.

### 3.2. Participants

A total of 145 first-year undergraduate university students participated in the study across three data collection points, representing a 75.13% response rate overall (time 1 = 49.22%, time 2 = 28.21%, and time 3 = 19.69%). Table 1 presents a breakdown of the sample characteristics by each data point (t1, t2, and t3). Ethnicity data were collected based on the United Kingdom Government-agreed list of ethnic groups used in the Census of England and Wales 2021 (GOV.UK, n.d.).

### 3.3. Participant Attrition

A total of 113 (77.9%) of all participants provided data at one data point, 21 (14.5%) provided data at two data points, and 11 (7.6%) provided data at all three data points. Of the 95 participants providing data at t1, 25 (26.31%) participated at t2, and 17 (17.89%) participated at t3. Thus, attrition was 73.69% between t1 and t2, 82.11% between t1 and t3, and 67.28% between t2 and t3. Participants who provided data at t1 and at least one further data collection point (i.e., t2 and/or t3) (*n* = 64) were compared with participants who provided data at t1 only (*n* = 31) on baseline characteristics and measures. Independent-samples *t*-tests and chi-square analyses indicated no significant differences in age, gender, ethnicity, program of study, or baseline sense of belonging, involvement, or engagement (*p* > 0.05), indicating that participant attrition was unlikely to have caused major bias.

### 3.4. Psychometric Measures

#### 3.4.1. Departmental Sense of Belonging and Involvement (DeSBI) (Knekta et al., 2020)

The DeSBI is a questionnaire-based measure of student sense of belonging and student involvement specifically relating to their ‘home’ department. ‘Home’ refers to the department where the students’ academic program is based and with which the student has most interaction. In the case of the present study, this is the ‘Psychology Directorate’. The DeSBI comprises three subscales, *sense of belonging: valued competence* (5 items), *sense of belonging: social acceptance* (6 items), and *involvement* (9 items). All items are positively worded. Students respond to each item along a 5-point Likert scale from strongly disagree (1) to strongly agree (5) so that higher scores indicate greater sense of belonging and involvement. While the DeSBI was originally developed using a 6-point Likert response scale, a 5-point scale was used in the present study to ensure structural consistency across the wider survey, which included several other measures that utilize a 5-point response format. Although arguably limiting direct comparison with existing normative data for the DeSBI, standardizing the response format has the advantage of mitigating forced choice bias and systematic measurement error, and reducing the risk of response errors and respondent fatigue. This is in line with suggested best practice in relation to optimum number of response categories (Gehlbach & Artino, 2018; Nielsen et al., 2017; Taufique et al., 2026). Similar response format modifications are evident in the psychometric literature (e.g., Nielsen et al., 2017), with evidence that altering the number of response categories, in this case from six to five, does not necessarily alter an instrument’s inherent psychometric properties (see Abulela & Khalaf, 2024 for a discussion). Example items include “People in the Psychology Directorate notice when I am good at something” (i.e., valued competence), “I am treated with as much respect as other students” (i.e., social acceptance), “During this academic year, it is likely that I will attend a seminar hosted by the Psychology Directorate” (i.e., involvement). Knekta et al. (2020) report internal reliability (*α*) as 0.90, 0.89, and 0.89, respectively, for valued competence, social acceptance, and involvement. Based on their results, the authors of the DeSBI argue that while related to sense of belonging, involvement is a separate construct. As such, total DeSBI scores are based on the summed total of the eleven sense of belonging items, while the involvement subscale is treated as a separate measure of student involvement for the purposes of analysis.

#### 3.4.2. Imperial College London Sense of Belonging Scale (ICL-SoB) (E. Cohen & Viola, 2022)

The ICL-SoB is a 10-item measure of student sense of belonging in higher education developed at Imperial College London (E. Cohen & Viola, 2022). Students respond to questions along a 5-point Likert-type scale with specific verbal labels tailored to each question. For example, “How happy are you with your choice to be a student in the Psychology Directorate?” Not at all happy, Slightly Happy, Somewhat Happy, Quite Happy, Extremely Happy. Responses are scored from 1 (not at all) to 5 (extremely), such that higher scores indicate positive responses and greater sense of belonging. According to its authors, the ICL-SoB represents three aspects of belonging, *academic belonging* (4 items), *overall belonging* (3 items), and *interpersonal belonging* (3 items). Although E. Cohen and Viola (2022) do not provide details of the ICL-SoB’s psychometric properties, the associated webpage does state that the scale has “undergone a rigorous, research-based development process to enhance reliability” (Sense of Belonging Scale, 2026). Total ICL-SoB scores were calculated by summing responses to all ten questions, while subscale scores were calculated by summing responses to academic belonging, overall belonging, and interpersonal belonging questions separately.

#### 3.4.3. Brief Departmental Student Sense of Belonging Scale (Brief DSSoB)

The Brief Departmental Sense of Belonging Scale (Brief DSSoB) (see Supplementary Materials) is a 6-item measure of student sense of belonging. It was developed as part of the present study, co-produced by the research team and student research assistant, with a particular focus on issues facing minoritized students that may not be fully captured by existing sense of belonging measures. Example items include “I feel connected to the culture and values of the Psychology Directorate”, “I feel that I can relate to others in the Psychology Directorate through shared interests or personal characteristics”. Students respond along a 5-point Likert scale from strongly disagree (1) to strongly agree (5), with higher scores representing greater sense of belonging. The final six items were selected by the research team from a larger initial pool of items based on their uniqueness and relevance to minoritized students.

#### 3.4.4. Student Engagement Subscale (Yorke, 2016)

The student engagement subscale is a 6-item scale developed alongside belongingness and self-confidence subscales as part of a larger survey exploring student retention and success (Yorke, 2016). Students respond along a 5-point Likert-type scale from strongly disagree (1) to strongly agree (5), so higher scores represent greater engagement. Example items include “I try to make connections between what I learn from different parts of my program”. Yorke (2016) reports good internal reliability (*α* = 0.75 to 0.77) and some evidence of age and gender differences supporting the validity of the engagement subscale.

### 3.5. Procedure

Following a favorable opinion from the institutional ethics panel (ref. 1312), first-year students in the Directorate of Psychology were invited to participate in the study by completing the online survey hosted on the Jisc Online Survey platform. Recruitment, including participant incentive, was advertised via posters displayed in the Psychology Directorate, on the student virtual learning environment, and on the Psychology Directorate participant recruitment platform. Participants navigated to the participant information and gave informed consent before completing the survey. Participants completing the survey were offered the opportunity to enter a cash prize draw. Prize draw entry was hosted on a separate Jisc page, accessed via a link provided after completing the survey to maintain participant anonymity. Participants were instructed to generate a unique participant code so that responses could be matched across data points, and so that participants could request that their data be withdrawn up to one week after completing the survey. Recruitment calls and prize draws were repeated each trimester. The survey took approximately ten minutes to complete and was completed in the participants’ own time using a device of their choice (e.g., computer, phone, tablet) in a location of their choice. The first survey (time 1) opened midway through the first trimester (week eight) to allow students the opportunity to settle into the Directorate and form a basis from which to respond to the sense of belonging survey items. The survey was repeated midway through the second trimester (time 2, week 24), and at the end of the first academic year (time 3, week 35). The academic year for undergraduate students consists of two trimesters.

### 3.6. Analysis

Comparative analysis was carried out to establish if there were differences in sense of belonging, involvement, and engagement between non-minoritized and minoritized ethic student groups, and to establish the effect of time on any group differences and on the development of sense of belonging, student involvement, and student engagement. In line with the study’s dynamic cohort design where students were able join, leave, and re-join the study at different data collection points, this analysis was performed using linear mixed-effects modeling to account for random effects (i.e., participants) and clustered, unbalanced data. Correlational analysis and hierarchical multiple regression were used to explore the relationship between sense of belonging, student involvement, and student engagement, and to investigate involvement and engagement as predictors of sense of belonging.

## 4. Results

### 4.1. Psychometrics

Reliability analysis presented in Table 2 shows that all measures and subscales met or exceeded the commonly applied threshold of *α* ≥ 0.70 for acceptable internal consistency reliability (Hussey et al., 2025).

### 4.2. Comparative Analysis

Estimated marginal means and standard errors presented in Table 3 show some variation in sense of belonging, involvement, and engagement by both time and group. However, only differences in involvement and sense of belonging (measured using the ICL-SoB) by time, but not group, were significant. Sense of belonging and involvement declined after time 1. Linear mixed-effects model analysis (type III test of fixed effects) with fixed (time and group) and random (participants) effects was performed, confirming a significant effect of time on sense of belonging (ICL-SoB) (*F*(2, 93.785) = 3.753, *p* = 0.027), and involvement (*F*(2, 124.221) = 3.921, *p* = 0.022). Group and interaction effects were not significant (*p* > 0.05).

Post hoc pairwise comparisons with Bonferroni correction performed for sense of belonging ICL-SoB did not identify a significant difference between time 1 (*EMM* = 35.84, *SE* = 0.66) and time 2 (*EMM* = 33.93, *SE* = 0.82) (mean difference = 1.908, *p* = 0.104, 95% CI [−0.262, 4.078]), time 1 (*EMM* = 35.84, *SE* = 0.66) and time 3 (*EMM* = 33.48, *SE* = 0.96) (mean difference = 2.363, *p* = 0.07, 95% CI [−0.138, 4.864]), or time 2 (*EMM* = 33.93, *SE* = 0.82) and time 3 (*EMM* = 33.48, *SE* = 0.96) (mean difference = 0.455, *p* = 1.0, 95% CI [−2.282, 3.192]).

Post hoc pairwise comparisons with Bonferroni correction performed for involvement did identify a significant difference between time 1 (*EMM* = 31.52, *SE* = 0.65) and time 2 (*EMM* = 28.92, *SE* = 0.82) (mean difference = 2.601, *p* = 0.02, 95% CI [0.314, 4.888]). Differences between time 1 (*EMM* = 31.52, *SE* = 0.65) and time 3 (*EMM* = 30.08, *SE* = 0.98) (mean difference = 1.436, *p* = 0.562, 95% CI [−1.192, 4.065]), and between time 2 (*EMM* = 28.92, *SE* = 0.82) and time 3 (*EMM* = 30.08, *SE* = 0.98) (mean difference = 1.165, *p* = 0.985, 96% CI [−4.045, 1.715]) were not significant.

### 4.3. Comparative Analysis by Subscale

Table 4 presents summary statistics for the subscales of the DeSBI and ICL-SoB sense of belonging measures. Despite some evidence of variation in estimated marginal means and standard errors by group and time, only differences in academic belonging by time, but not group, were found to be significant. Linear mixed-effects model analysis (type III test of fixed effects) with fixed (time and group) and random (participants) effects confirmed a significant effect for time on academic belonging (*F*(2, 96.91) = 4.677, *p* = 0.012). Group and interaction effects were not significant (*p* = 0.825, *p* = 0.184).

Post hoc pairwise comparisons with Bonferroni correction performed for academic belonging identified a significant difference between time 1 (*EMM* = 15.87, *SE* = 0.261) and time 3 (*EMM* = 14.78, *SE* = 0.384) (mean difference = 1.397, *p* = 0.029, 95% CI [0.083, 2.104]). Differences between time 1 (*EMM* = 15.87, *SE* = 0.261) and time 2 (*EMM* = 15.04, *SE* = 0.327) mean difference = 0.833, *p* = 0.068, 95% CI [−0.044, 1.710]), and between time 2 (*EMM* = 15.04, *SE* = 0.327) and time 3 (*EMM* = 14.78, *SE* = 0.384) (mean difference—0.260, *p* = 1.0, 95% CI [−0.846, 1.367]) were not significant.

### 4.4. Correlational Analysis

Correlational analysis (Table 5, Table 6 and Table 7) revealed significant positive correlations (effect size (*ES*) = medium to large, J. Cohen, 1988) between sense of belonging (all measures) and both involvement and engagement at all time points (t1, t2, & t3), with the exception of DeSBI and involvement at time 2, where the correlation was non-significant (*p* > 0.05). Positive correlations between sense of belonging measures (DeSBI, ICL-SoB, and Brief DSSoB) at all time points were significant (*ES* = large).

### 4.5. Correlational Analysis by Subscale

Correlational analysis (Table 8, Table 9 and Table 10) revealed significant positive correlations between all sense of belonging subscales at all time points (t1, t2, & t3) (*ES* = large), with the exception of valued competence and overall belonging (t3), between all subscales and engagement at all time points (*ES* = medium to large) and between all subscales and involvement at all time points (*ES* = medium to large), with the exception social acceptance (t2 & t3) and overall belonging (t2).

### 4.6. Regression Analysis

Hierarchical multiple linear regression was conducted to determine if, after controlling for age and gender (model 1), student engagement (model 2) and student involvement (model 3) predicted student sense of belonging. Separate analyses were performed for each sense of belonging measure and their subscales. Analysis was performed on trimester one (t1) data only, where the sample size was considered sufficient to support the reliability of the regression models involving two predictors (e.g., Green, 1991). Collinearity was not a concern, with all predictor variables falling below the VIF threshold of 3 and exceeding the tolerance of 0.1.

Regression models predicting student sense of belonging from student involvement and student engagement controlling for age and gender were significant for each of the sense of belonging measures and associated subscales, explaining between 30.5% and 40.1% and 25.8% to 43.2% of variance respectively: DeSBI 36.5% (*F*(4, 88) = 12.658, *R*^2^ = 0.365, *p* < 0.001); ICL-SoB 40.1% (*F*(4, 88) = 14.706, *R*^2^ = 0.401, *p* < 0.001); Brief DSSoB 30.5% (*F*(4, 88) = 9.633, *R*^2^ = 0.305, *p* < 0.001); social acceptance subscale (DeSBI) 25.8% (*F*(4, 88) = 7.647, *R*^2^ = 0.258, *p* < 0.001); valued competence subscale (DeSBI) 34.3% (*F*(4, 88) = 11.510, *R*^2^ = 0.343, *p* < 0.001); academic belonging subscale (ICL-SoB) 29% (*F*(4, 88) = 9.004, *R*^2^ = 0.290, *p* < 0.001); interpersonal belonging subscale (ICL-SoB) 43.2% (*F*(4, 88) = 16.748, *R*^2^ = 0.432, *p* < 0.001); and overall belonging subscale (ICL-SoB) 34.6% (*F*(4, 88) = 11.655, *R*^2^ = 0.346, *p* < 0.001). However, student involvement did not add significant unique predictive power to the models, with student engagement identified as the only significant predictor of sense of belonging in all models (see Table 11 and Table 12).

## 5. Discussion

Despite an evident increase in research exploring student sense of belonging in higher education—driven by evidence supporting its positive impact on critical outcomes, including being a negative predictor of students’ intention to leave (Allen et al., 2024; Crawford et al., 2024; Gilani & Thomas, 2025)—there has been limited attention given to ‘context’. The principal aim of the study was therefore to gain a better understanding of context-specific, *situated*, student sense of belonging in higher education, with students’ home department serving as the primary context for measurement. Differences in student sense of belonging, student involvement, and student engagement according to ethnicity and time were explored in first-year university students. Student involvement and engagement were also explored as salient factors in student sense of belonging.

Comparing minoritized ethnic students with non-minoritized ethnic students, no significant differences in student sense of belonging, student involvement, or student engagement were found. The lack of significant effect of student ethnicity is contrary to previous studies reporting lower sense of belonging in minoritized ethnic students (Dias-Broens et al., 2024; Kim & Lundberg, 2026; Yorke, 2016). Findings also contradict evidence suggesting minoritized students may struggle to belong (Allen et al., 2021) due to difficulties experienced with social integration, lack of friends on the same course, and peer support, where culture was a “hindrance” and feeling “different” to the majority was reported (Kauser et al., 2021, p. 15). That differences in student sense of belonging were not found may highlight the critical importance of context and the value of evidence from situated home department studies. Previous studies and measures have largely focused on the institution as the primary context. That is, whether students report a sense of belonging to the wider school, college, or university. However, sense of belonging has been described as situational and context-dependent (Allen et al., 2021; Dias-Broens et al., 2024; Kern et al., 2020), reliant on peer relationships, staff-student interactions, knowledge, confidence and identity, and relevance to student interests and future goals (Thomas, 2012). It is the students’ home department, then, we argue, that provides the most meaningful and valid context in which to capture sense of belonging in higher education. This is where the student is ‘situated’ and provides the greatest opportunities for meaningful peer and staff interactions, learning and gaining knowledge, and developing confidence and identity related to students’ interests and goals at university (Knekta et al., 2020). In the case of the present study, the students’ department seems to offer similar opportunities to both minoritized ethnic and non-minoritized ethnic students for developing sense of belonging. Findings may have been different if the study was situated in the context of the wider institution rather than the department. This is an important distinction if research evidence is used to inform decisions regarding changes in policy and practice, where making assumptions based on evidence from studies conducted in contexts or situations that are less directly relevant could lead to ineffective or counterproductive changes.

That no significant difference in involvement or engagement was found when comparing minoritized ethnic and non-minoritized ethnic students aligns with previous research, provided that we accept student involvement as similar in nature to the behavioral dimension of engagement (Maroco et al., 2016; Otermans et al., 2025, Sun et al., 2024). Otermans et al. (2025), for example, reported no difference according to ethnicity in behavioral, cognitive, or emotional engagement. Furthermore, the consistent lack of difference between minoritized and non-minoritized ethnic students in sense of belonging, student involvement, and student engagement supports the reported close association between these three constructs (Osterman, 2000; Strayhorn, 2019; Thomas, 2012).

The study’s findings revealed significant differences in student sense of belonging and student involvement, but not student engagement. Both students’ sense of belonging and student involvement declined during students’ first year. These findings were anticipated and are supported by previous studies presenting evidence of a general decline in sense of belonging, specifically during their first year (Gilani et al., 2026; Hausmann et al., 2007), a decline in student involvement/behavioral engagement (Bognar & Khine, 2025; Boulton et al., 2019), and stable student engagement (Korhonen et al., 2024; Saqr & Lopez-Pernas, 2021). In their study, Brown and Papp (2025) measured sense of belonging in the context of the wider campus community, explaining the decline in terms of a process of students’ ‘de-identification’ with the wider campus community in favor of increased identification with their department as they advance in their academic studies. That students report a similar decline in situated sense of belonging measured in the context of their home department suggests the need for an alternative explanation not reliant on the interplay of university campus versus department identity salience. Allen et al. (2024) argue that personal development and changes in the environment experienced at university will influence students’ sense of belonging so that it evolves over time, possibly explaining changes in sense of belonging irrespective of context. Other explanations for a decline in sense of belonging include initial artificial inflation due to universities’ investment in supportive student induction and onboarding early in the first year. These initiatives are not maintained, giving way to the reality of the challenges of academic life and separation from previous, well-established, support systems (Gilani et al., 2026). In their study, Gilani et al. (2026) note that differences in sense of belonging only reached significance when comparing the beginning of the year to the end of the year and suggested that this may reflect cumulative effects of academic-related stress, fatigue, and isolation from staff and peers as the academic year finishes and the engagement and support provided at the beginning of the year, when sense of belonging was higher, have dissipated. This is consistent with findings from the present study, where differences in sense of belonging were only identified between the beginning and end of the year (t1 & t3).

In the present study, differences in student sense of belonging were only evident when measured using the D-SoB, with subscale analysis only identifying significant differences in academic belonging. No significant differences according to time were identified for the valued competence, social acceptance, interpersonal belonging, or overall belonging subscales. This suggests, first, that all measures of student sense of belonging are not, unsurprisingly, the same, and that researchers should consider carefully the selection of appropriate instruments in future studies. Some measures, such as the ICL-SoB, may be more sensitive to temporal variation, which may be a priority for some studies but not others. Secondly, that academic belonging, but not interpersonal or other aspects of belonging, accounts for the decline in students’ sense of belonging helps support Nunn’s (2021) account of social, academic, and campus-community belonging as independent aspects of belonging that do not necessarily influence each other. It also aligns with findings from previous studies that, unlike interpersonal/social belonging which gradually gets stronger, academic belonging (i.e., students’ perception of fit and connection to their chosen discipline and academic program; respected by staff and students, enriching experience, and choice of department) fluctuates during the first year of university (Kahu et al., 2022, p. 15). That it is academic belonging that appears to account for the decline in situated student sense of belonging suggests that this should be a primary focus for efforts intended to foster increased student sense of belonging. Indeed, Kahu et al. (2022, p. 15) found that academic belonging “mattered more [than other aspects of belonging] … without academic belonging to sustain them, workload and stress had a greater negative impact on students”, including a greater propensity to change course or drop out.

Though far from straightforward, findings from previous studies exploring temporal changes in student involvement and student engagement broadly support findings from the present study. Where there is a focus on behavioral or physical engagement or involvement measuring students’ tangible participation in learning and extracurricular activities, a decline across time is often reported (e.g., Boulton et al., 2019). Previous studies reporting a decline in student involvement suggest that this may be accounted for by students’ eagerness to ‘get ahead’ early in the year, the initial novelty of new experiences (which inevitably diminish as the year progresses), and timing and associated workload of assessments (Bognar & Khine, 2025; Boulton et al., 2019). That a significant decline in involvement was evident between the first and second trimester in the present study supports this explanation based on initial novelty, increasing workload, and timing of assessment. That student engagement remained stable likely reflects the focus on cognitive and emotional/interpersonal aspects of engagement in the present study, which are not subject to the same influences responsible for the decline in involvement or behavioral engagement. In studies where the focus is on cognitive and emotional/interpersonal (i.e., psychological) dimensions of engagement measuring “investment in learning [and] emotional responses in the context of learning and interaction”, student engagement remains stable across time (e.g., Korhonen et al., 2024, p. 2; Saqr & Lopez-Pernas, 2021).

The significant positive correlations between situated student sense of belonging and both involvement and engagement reported in the present study were expected and are supported by previous studies reporting a close association between these constructs (Osterman, 2000; Strayhorn, 2019; Thomas, 2012). However, only student engagement, not student involvement, was identified as a significant positive predictor of student sense of belonging. That engagement encompasses cognitive and emotional/interpersonal dimensions as well as a behavioral dimension, in contrast to involvement which has limited scope and a transient nature focusing on behavioral and physical aspects of engagement, may account for its status as a predictor of student sense of belonging. Adding involvement to regression models predicting sense of belonging did not account for any significant additional variance beyond that accounted for by student engagement. These findings help support previous suggestions that involvement should be considered an aspect or dimension of engagement rather than a separate construct (Maroco et al., 2016; Otermans et al., 2025, Sun et al., 2024). Furthermore, while the relationship between student engagement and student sense of belonging has been described as “two-way directional” (Gilani & Thomas, 2025, p. 19), studies have largely evidenced the influence of student sense of belonging on student engagement (e.g., Gillen-O’Neel, 2021), signaling the need for studies, such as the present study, focusing on the influence student engagement may have on student sense of belonging (Gilani & Thomas, 2025). Although not in itself evidence of a causal association, the emergence of student engagement as a predictor of first-year students’ sense of belonging in the present study adds to existing evidence supporting the view that engagement activities “*give*” students a sense of belonging (Humphrey & Lowe, 2017, p. 185), and “engagement to promote belonging must begin early and continue across the student life cycle” (Thomas, 2012, p. 16).

### Limitations and Future Directions

The study has several limitations and associated future directions for research that should be considered. Although the study’s dynamic cohort design helped mitigate high participant attrition typical in longitudinal studies and maintain adequate sample size, the approach increases participant variation. While analysis strategies were selected to address this issue, larger-scale closed cohort studies will help confirm findings from the present study and preserve sufficient statistical power for pairwise comparisons to improve the detection of meaningful differences. The study set out clearly its approach to assigning students to minoritized ethnic and non-minoritized ethnic student groups. However, there are alternative approaches, and this should be considered when interpreting findings and planning future studies. Although each of the selected psychometric measures has merit and the study provided promising evidence supporting their psychometric properties, including the newly developed Brief DSSoB, there is a need for further work to evidence their validity and reliability. This is particularly the case in respect of the Brief DSSoB, which has so far been subjected to limited reliability and validity testing and therefore exists as a preliminary measure. This relates to a broader issue around the need for consensus regarding agreed conceptual definitions and valid and reliable construct measures for each of the key constructs investigated (i.e., student sense of belonging, student involvement, and student engagement). Future studies that include separate sense of belonging measures situated in the contexts of both the department and the wider institution (e.g., university) in a single diverse sample of students with multiple data points will provide evidence regarding our assertion that sense of belonging is situated, as well as Brown and Papp’s (2025) de-identification hypothesis used to explain context-specific decline in sense of belonging. Such studies will also provide evidence regarding whether differences in sense of belonging between minoritized and non-minoritized ethnic students are context-dependent, as findings from the present study suggest. Finally, the study did not address intersectionality. Students’ sense of belonging is likely subject to the effects of overlapping social identities, an area warranting further investigation in higher education studies.

## 6. Conclusions

Contrary to much of the previous evidence examining sense of belonging in the context of the wider campus or university, no difference in sense of belonging between minoritized and non-minoritized ethnic students was identified in the context of students’ home department. While not conclusive, this serves as evidence supporting sense of belonging as context-specific and situated, highlighting the potential for university departments to address issues of equality in ways that the wider institution may not be able to. As reported in previous studies, student sense of belonging, in particular academic belonging, declined during the first year according to some, but not all, psychometric measures used in the present study. Thus, department efforts to develop student sense of belonging in the early stages of the students’ university experience must be maintained, even enhanced, throughout the year and beyond, with a focus on academic belonging. Such efforts should include meaningful and positive staff and student interactions, recognition of students’ achievements, values, and identities, opportunities for students to contribute and influence departmental activities, and opportunities for students to connect with their discipline and department. Staff/student co-creation initiatives, staff/student forums, peer mentoring programs, discipline-specific staff/student learning communities, staff/student research and curriculum development projects, discipline-specific departmental seminars, discipline-specific student-led workshops, seminars, conferences, and societies, connecting with alumni, and content-based classroom activities all serve as opportunities for students to engage. Evaluating the impact of these opportunities on belonging is also important. Given the evidence presented here, selecting the appropriate sense of belonging measure is critical, particularly if capturing change is a priority, as some measures may be more sensitive to change than others. Finally, findings from the present study identified student engagement as a significant predictor of situated student sense of belonging. Providing students with opportunities to engage is therefore of primary importance for higher education institutions. While involvement did not account for any additional variance in student sense of belonging beyond that accounted for by engagement, it should not be overlooked. Engagement, we argue, encompasses involvement in the form of student behaviors, cognitions, affective responses and emotions, and social interactions, and that the best place to provide opportunities for students to engage in these ways may well be in the students’ own department.

## Figures and Tables

**Table 1:** Sample characteristics by time and group.

Time	Group	*N*	Mean Age (*SD*)	Program (Single/Joint Honors)	Gender	Ethnicity
t1	Combined	95	21.52 (7.14)	Single 52.6% Joint 47.4%	Female 83.2% Male 16.8%	White British 54.6%, White Irish 1.1%, White other 14.7%, mixed-race 3.3%, Asian 14.7%, Black 11.6%
	White British	52	20.69 (5.04)	Single 42.3% Joint 57.7%	Female 84.6% Male 15.4%	White British 100%
	Minoritized Ethnic	43	22.56 (9.12)	Single 65.1% Joint 34.9%	Female 81.4% Male 18.6%	White Irish 2.3%, White other 32.6%, mixed-race 7.0%, Asian 32.6%, Black 25.5%
t2	Combined	55	22.96 (9.91)	Single 60% Joint 40%	Female 81.8% Male 18.2%	White British 49.1%, White other 16.4%, mixed-race 1.8%, Asian 21.8%, Black 10.9%
	White British	27	20.96 (5.0)	Single 63% Joint 37%	Female 77.8% Male 22.2%	White British 100%
	Minoritized Ethnic	28	24.89 (11.26)	Single 57.1% Joint 42.9%	Female 85.7% Male 14.3%	White other 32.1%, mixed-race 3.6%, Asian 42.9%, Black 21.4%
t3	Combined	38	24.16 (9.1)	Single 44.7% Joint 55.3%	Female 86.8% Male 10.5%, Prefer Not to Say 2.6%	White British 47.4%, White other 18.4%, Asian 21.0%, Black 13.2%
	White British	18	21.28 (4.34)	Single 38.9% Joint 61.1%	Female 94.4% Male 5.6%	White British 100%
	Minoritized Ethnic	20	26.75 (11.31)	Single 50% Joint 50%	Female 80% Male 15% Prefer Not to Say 5%	White other 35.0%, Asian 40.0%, Black 25.0%

**Table 2:** Reliability analysis.

Scale/Subscale	No. of Items	Theoretical Range	Cronbach’s Alpha
DeSBI	11	11–55	0.85
- Valued Competence	5	5–25	0.86
- Social Acceptance	6	6–30	0.82
- Involvement	9	9–45	0.84
ICL-SoB	10	10–50	0.89
- Academic Belonging (AB)	4	4–20	0.73
- Interpersonal Belonging (IB)	3	3–15	0.76
- Overall Belonging (OB)	3	3–15	0.81
Brief DSSoB	6	6–30	0.89
Engagement	6	6–30	0.85

**Table 3:** Estimated marginal means (standard error) [confidence interval (95%)] for student sense of belonging by time, ethnicity, and sense of belonging measure.

Measure	Group	Time 1	Time 2	Time 3
DeSBI	White British	40.87 (0.87) [39.16, 42.59] *N* = 52	38.92 (1.17) [36.61, 41.22] *N* = 27	39.48 (1.38) [36.75, 42.21] *N* = 18
	Minoritized Ethnic	40.61 (0.95) [38.74, 42.48] *N* = 43	40.78 (1.14) [38.53, 43.02] *N* = 28	39.88 (1.34) [37.23, 42.53] *N* = 20
ICL-SoB *	White British	35.78 (0.89) [34.02, 37.54] *N* = 52	32.77 (1.17) [30.46, 35.09] *N* = 27	33.95 (1.36) [31.25, 36.64] *N* = 18
	Minoritized Ethnic	35.90 (0.97) [33.99, 37.81] *N* = 43	35.09 (1.14) [32.84, 37.34] *N* = 28	33.01 (1.34) [30.36, 35.66] *N* = 20
Brief DSSoB	White British	22.94 (0.60) [21.76, 24.13] *N* = 52	21.55 (0.78) [20.00, 23.09] *N* = 27	21.76 (0.90) [19.98, 23.55] *N* = 18
	Minoritized Ethnic	22.89 (0.65) [21.60, 24.17] *N* = 43	22.75 (0.76) [21.26, 24.25] *N* = 28	22.22 (0.89) [20.46, 23.98] *N* = 20
Involvement *	White British	31.10 (0.88) [29.38, 32.83] *N* = 52	27.36 (1.18) [25.03, 29.69] *N* = 27	30.78 (7.54) [27.26, 32.80] *N* = 18
	Minoritized Ethnic	31.93 (0.95) [30.0, 33.82] *N* = 43	30.47 (1.15) [28.20, 32.74] *N* = 28	30.34 (1.40) [27.26, 32.80] *N* = 20
Engagement	White British	23.81 (0.48) [22.87, 24.75] *N* = 52	23.69 (0.62) [22.46, 24.92] *N* = 27	24.88 (0.72) [23.45, 26.31] *N* = 18
	Minoritized Ethnic	23.97 (0.52) [22.94, 24.99] *N* = 43	23.83 (0.61) [22.64, 25.03] *N* = 28	22.59 (0.71) [21.19, 24.00] *N* = 20

* *p* < 0.05.

**Table 4:** Estimated marginal means (standard error) [confidence interval (95%)] for student sense of belonging by time, ethnicity, and subscale.

Subscale	Group	Time 1	Time 2	Time 3
Valued Competence	White British	17.27 (0.49) [16.30, 18.25] *N* = 52	15.90 (0.68) [14.56, 17.24] *N* = 27	18.50 (0.82) [15.88, 19.12] *N* = 18
	Minoritized Ethnic	16.90 (0.54) [15.83, 17.98] *N* = 43	17.56 (0.66) [16.25, 18.87] *N* = 28	17.42 (0.79) [15.87, 18.98] *N* = 20
Social Acceptance	White British	23.66 (0.55) [22.58, 24.73] *N* = 52	23.06 (0.73) [21.63, 24.50] *N* = 27	22.02 (0.86) [20.33, 23.72] *N* = 18
	Minoritized Ethnic	23.70 (0.59) [22.53, 24.87] *N* = 43	23.18 (0.71) [21.78, 24.58] *N* = 28	22.49 (0.84) [20.84, 24.14] *N* = 20
Academic Belonging *	White British	15.75 (0.35) [15.05, 16.45] *N* = 52	14.76 (0.47) [13.85, 15.70] *N* = 27	15.32 (0.55) [14.24, 16.40] *N* = 18
	Minoritized Ethnic	16.00 (0.39) [15.24, 16.76] *N* = 43	15.31 (0.46) [14.81, 16.20] *N* = 28	14.24 (0.54) [13.18, 15.30] *N* = 20
Interpersonal Belonging	White British	10.03 (0.30) [9.44, −10.63] *N* = 52	8.83 (0.40) [8.03, 9.63] *N* = 27	9.40 (0.48) [8.45, 10.35] *N* = 18
	Minoritized Ethnic	9.80 (0.33) [9.14, 10.44] *N* = 43	9.61 (0.39) [8.84, 10.39] *N* = 28	9.58 (0.47) [8.66, 10.50] *N* = 20
Overall Belonging	White British	11.02 (0.35) [10.34, 11.71] *N* = 52	10.16 (0.46) [9.26, 11.07] *N* = 27	10.77 (0.53) [9.71, 11.82] *N* = 18
	Minoritized Ethnic	11.17 (0.38) [10.42, 11.91] *N* = 43	10.50 (0.44) [9.63, 11.38] *N* = 28	10.20 (0.52) [9.17, 11.24] 20

* *p* < 0.05.

**Table 5:** Correlation coefficients [95% CI] for sense of belonging, involvement, and engagement (time 1).

	ICL-SoB	Brief DSSoB	Involvement	Engagement
DeSBI	0.76 *** [0.68, 1.0] *N* = 95	0.78 *** [0.70, 1.0] *N* = 95	0.46 *** [0.31, 1.0] *N* = 95	0.59 *** [0.46, 1.0] *N* = 95
ICL-SoB	-	0.71 *** [0.62, 1.0] *N* = 95	0.48 *** [0.34, 1.0] *N* = 95	0.63 *** [0.51, 1.0] *N* = 95
Brief DSSoB	-	-	0.31 *** [0.15, 1.0] *N* = 95	0.54 *** [0.41, 1.0] *N* = 95
Involvement	-	-	-	0.62 *** [0.51, 1.0] *N* = 95

*** *p* < 0.001 (1-tailed).

**Table 6:** Correlation coefficients [95% CI] for sense of belonging, involvement, and engagement (time 2).

	ICL-SoB	Brief DSSoB	Involvement	Engagement
DeSBI	0.76 *** [0.64, 1.0] *N* = 55	0.86 *** [0.79, 1.0] *N* = 55	0.17 [−0.06, 1.0] *N* = 55	0.35 ** [0.14, 1.0] *N* = 55
ICL-SoB	-	0.76 *** [0.65, 1.0] *N* = 55	0.31 ** [0.95, 1.0] *N* = 55	0.53 *** [0.35, 1.0] *N* = 55
Brief DSSoB	-	-	0.27 * [0.04, 1.0] *N* = 55	0.44 *** [0.24, 1.0] *N* = 55
Involvement	-	-	-	0.62 *** [0.46, 1.0] *N* = 55

* *p* < 0.05, ** *p* < 0.01, *** *p* < 0.001 (1-tailed).

**Table 7:** Correlation coefficients [95% CI] for sense of belonging, involvement, and engagement (time 3).

	ICL-SoB	Brief DSSoB	Involvement	Engagement
DeSBI	0.64 *** [0.44, 1.0] *N* = 38	0.82 *** [0.70, 1.0] *N* = 38	0.40 ** [0.14, 1.0] *N* = 38	0.41 ** [0.16, 1.0] *N* = 38
ICL-SoB	-	0.67 *** [0.49, 1.0] *N* = 38	0.36 * [0.10, 1.0] *N* = 38	0.55 *** [0.33, 1.0] *N* = 38
Brief DSSoB	-	-	0.43 ** [0.18, 1.0] *N* = 38	0.56 *** [0.34, 1.0] *N* = 38
Involvement	-	-	-	0.41 ** [0.16, 1.0] *N* = 38 *

* *p* < 0.05, ** *p* < 0.01, *** *p* < 0.001 (1-tailed).

**Table 8:** Correlation coefficients [95% CI] for sense of belonging subscales, involvement, and engagement (time 1).

	VC	AB	IB	OB	Involvement	Engagement
Social Acceptance	0.40 *** [0.25, 1.0] *N* = 95	0.59 *** [0.47, 1.0] *N* = 95	0.44 *** [0.29. 1.0] *N* = 95	0.45 *** [0.31, 1.0] *N* = 95	0.29 ** [0.12, 1.0] *N* = 95	0.44 *** [0.30, 1.0] *N* = 95
Valued Competence	-	0.57 *** [0.44, 1.0] *N* = 95	0.66 *** [0.56, 1.0] *N* = 95	0.56 *** [0.43, 1.0] *N* = 95	0.48 *** [0.34, 1.0] *N* = 95	0.54 *** [0.41, 1.0] *N* = 95
Academic Belonging	-	-	0.59 *** [0.47, 1.0] *N* = 95	0.74 *** [0.65, 1.0] *N* = 95	0.40 *** [0.24, 1.0] *N* = 95	0.52 *** [0.39, 1.0] *N* = 95
Interpersonal Belonging	-	-	-	0.60 *** [0.48, 1.0] *N* = 95	0.55 *** [0.42, 1.0] *N* = 95	0.62 *** [0.50, 1.0] *N* = 95
Overall Belonging	-	-	-	-	0.43 *** [0.28, 1.0] *N* = 95	0.59 *** [0.47, 1.0] *N* = 95

** *p* < 0.01, *** *p* < 0.001 (1-tailed).

**Table 9:** Correlation coefficients [95% CI] for sense of belonging sub-scales, involvement, engagement, and age (time 2).

	VC	AB	IB	OB	Involvement	Engagement
Social Acceptance	0.52 *** [0.34, 1.0] *N* = 55	0.58 *** [0.41, 1.0] *N* = 55	0.26 * [0.04, 1.0] *N* = 55	0.67 *** [0.52, 1.0] *N* = 55	−0.02 [−1.0, 0.20] *N* = 55	0.24 * [0.01, 1.0] *N* = 55
Valued Competence	-	0.48 *** [0.29, 1.0] *N* = 55	0.58 *** [0.41, 1.0] *N* = 55	0.64 *** [0.49, 1.0] *N* = 55	0.35 ** [0.13, 1.0] *N* = 55	0.40 *** [0.19, 1.0] *N* = 55
Academic Belonging	-	-	0.52 *** [0.33, 1.0] *N* = 55	0.72 *** [0.60, 1.0] *N* = 55	0.23 * [0.01, 1.0] *N* = 55	0.44 *** [0.24, 1.0] *N* = 55
Interpersonal Belonging	-	-	-	0.54 *** [0.36, 1.0] *N* = 55	0.68 *** [0.54, 1.0] *N* = 55	0.61 *** [0.45, 1.0] *N* = 55
Overall Belonging	-	-	-	-	0.16 [−0.06, 1.0] *N* = 55	0.41 *** [0.20, 1.0] *N* = 55

* *p* < 0.05, ** *p* < 0.01, *** *p* < 0.001 (1-tailed).

**Table 10:** Correlation coefficients [95% CI] for sense of belonging sub-scales, involvement, engagement, and age (time 3).

	VC	AB	IB	OB	Involvement	Engagement
Social Acceptance	0.34 * [0.08, 1.0] *N* = 38	0.64 *** [0.44, 0.80] *N* = 38	0.40 ** [0.14, 1.0] *N* = 38	0.66 *** [0.48, 1.0] *N* = 38	0.23 [−0.05, 1.0] *N* = 38	0.34 * [0.07, 1.0] *N* = 38
Valued Competence	-	0.28 * [0.01, 1.0] *N* = 38	0.49 *** [0.25, 1.0] *N* = 38	0.24 [−0.03, 1.0] *N* = 38	0.45 ** [0.20, 1.0] *N* = 38	0.35 * [0.08, 1.0] *N* = 38
Academic Belonging	-	-	0.58 *** [0.36, 1.0] *N* = 38	0.78 *** [0.64, 1.0] *N* = 38	0.28 * [0.01, 1.0] *N* = 38	0.48 *** [0.23, 1.0] *N* = 38
Interpersonal Belonging	-	-	-	0.60 *** [0.39, 1.0] *N* = 38	0.62 *** [0.42, 1.0] *N* = 38	0.36 * [0.10, 1.0] *N* = 38
Overall Belonging	-	-	-	-	0.30 * [0.03, 1.0] *N* = 38	0.62 *** [0.42, 1.0] *N* = 38

* *p* < 0.05, ** *p* < 0.01, *** *p* < 0.001 (1-tailed).

**Table 11:** Regression analysis showing age, gender, engagement, and involvement as predictors of student sense of belonging by sense of belonging measure.

Measure/ Variable	Cumulative			Simultaneous			
	*R*^2^ Change	*F*-Change		*B*	β	*t*	*p*
**DeSBI**							
*Model 1*	0.009	*F*(2, 90) = 0.426, *p* = 0.654					
Age				−0.134	−0.151	−1.709	0.091
Gender				1.241	0.075	0.866	0.389
*Model 2*	0.340	*F*(1, 89) = 46.537, *p* < 0.001					
Engagement				0.801	0.494	4.595	<0.001
*Model 3*	0.016	*F*(1, 88) = 2.178, *p* = 0.144					
Involvement				0.174	0.162	1.476	0.144
**ICL-SoB**							
*Model* 1	0.005	*F*(2, 90) = 0.212, *p* = 0.809					
Age				−0.039	−0.043	−0.497	0.620
Gender				−0.150	−0.009	−0.104	0.917
*Model 2*	0.381	*F*(1, 89) = 55.194, *p* < 0.001					
Engagement				0.892	0.530	5.080	<0.001
*Model 3*	0.015	*F*(1, 88) = 2.199, *p* = 0.142					
Involvement				0.176	0.158	1.483	0.142
**Brief DSSoB**							
*Model 1*	0.005	*F*(2, 90) = 0.249, *p* = 0.780					
Age				−0.076	−0.127	−1.375	0.173
Gender				−0.560	−0.050	−0.556	0.579
*Model 2*	0.299	*F*(1, 89) = 38.191, *p* < 0.001					
Engagement				0.618	0.567	5.048	<0.001
*Model 3*	0.000	*F*(1, 88) = 0.053, *p* = 0.818					
Involvement				−0.019	−0.026	−0.230	0.818

**Table 12:** Regression analysis showing age, gender, engagement, and involvement as predictors of sense of belonging by sense of belonging subscale.

Measure/ Variable	Cumulative			Simultaneous			
	*R*^2^ Change	*F*-Change		*B*	β	*t*	*p*
**Social Acceptance**							
*Model 1*	0.045	*F* (2, 90) = 2.103, *p* = 0.128					
Age				−0.151	−0.281	−2.941	0.004
Gender				−0.208	−0.021	−0.221	0.825
*Model 2*	0.211	*F*(1, 89) = 25.285, *p* < 0.001					
Engagement				0.425	0.431	3.711	<0.001
*Model 3*	0.002	*F*(1, 88) = 0.228, *p* = 0.634					
Involvement				0.037	0.057	0.478	0.634
**Valued Competence**							
*Model 1*	0.053	*F* (2, 90) = 2.510, *p* = 0.087					
Age				0.017	0.033	0.368	0.714
Gender				1.449	0.147	1.678	0.097
*Model 2*	0.263	*F*(1, 89) = 34.201, *p* < 0.001					
Engagement				0.376	0.392	3.581	<0.001
*Model 3*	0.028	*F*(1, 88) = 3.715, *p* = 0.057					
Involvement				0.137	0.215	1.927	0.057
**Academic Belonging**							
*Model 1*	0.002	*F* (2, 90) = 0.108, *p* = 0.897					
Age				−0.041	−0.133	−1.207	0.231
Gender				0.220	0.032	0.354	0.724
*Model 2*	0.276	*F*(1, 89) = 34.040, *p* < 0.001					
Engagement				0.297	0.447	3.937	<0.001
*Model 3*	0.012	*F*(1, 88) = 1.480, *p* = 0.225					
Involvement				0.062	0.142	1.221	0.225
**Interpersonal Belonging**							
*Model 1*	0.047	*F*(2, 90) = 2.197, *p* = 0.117					
Age				0.027	0.090	1.070	0.288
Gender				0.062	0.011	0.133	0.894
*Model 2*	0.350	*F*(1, 89) = 51.668, *p* < 0.001					
Engagement				0.252	0.454	4.472	<0.001
*Model 3*	0.035	*F*(1, 88) = 5.498, *p* = 0.021					
Involvement				0.089	0.244	2.345	0.021
**Overall Belonging**							
*Model 1*	0.011	*F*(2, 90) = 0.501, *p* = 0.608					
Age				0.003	0.008	0.089	0.929
Gender				0.002	0.000	0.004	0.097
*Model 2*	0.330	*F*(1, 89) = 44.521, *p* < 0.001					
Engagement				328	0.523	4.801	<0.001
*Model 3*	0.006	*F*(1, 88) = 0.745, *p* = 0.390					
Involvement				0.040	0.096	0.863	0.390

## Data Availability

The data presented in this study are available on request from the corresponding author.

## Supplementary Materials

The following supporting information can be downloaded at: https://www.mdpi.com/article/10.3390/bs16101809/s1, Table S1. Brief Departmental Student Sense of Belonging Scale.
