# Supplementary material for: Situated Student Sense of Belonging in Higher Education: The Importance of Context and Engagement
Source: Behav Sci (Basel). 2026 Oct 2;16(10):1809. doi: 10.3390/bs16101809 (PMC13701320; doi:10.3390/bs16101809)
Supplement: Supplementary file 1 [file behavsci-16-01809-s001.zip › behavsci-4533219-supplementary.pdf]

Supplementary Table S1. Brief Departmental Student Sense of Belonging Scale.

Please rate your agreement with the following statements based on how you feel about the [insert department name]:

Strongly Disagree **(SD)** Disagree **(D)** Neither Agree nor Disagree **(N)** Agree **(A)** Strongly Agree **(SA)**

	SD	D	N	A	SA
I feel comfortable asking for help from staff and students in the [insert department name]					
I feel connected to the culture and values of the [insert department name]					
I feel secure in the relationships I’ve built within the [insert department name]					
I feel comfortable sharing my thoughts and opinions with others in the [insert department name]					
I feel that I can relate to others in the [insert department name] through shared interests or personal characteristics.					
I feel a sense of happiness and contentment when I’m around people in the [insert department name]					
